# Presence of Tetracycline and Sulfonamide Resistance Genes in *Salmonella* spp.: Literature Review

**DOI:** 10.3390/antibiotics10111314

**Published:** 2021-10-28

**Authors:** Sabrina Lunara Santos Pavelquesi, Ana Carolina Almeida de Oliveira Ferreira, Angeislenie Ricelle Magalhães Rodrigues, Calliandra Maria de Souza Silva, Daniela Castilho Orsi, Izabel Cristina Rodrigues da Silva

**Affiliations:** Laboratory of Food Control, University of Brasilia (UnB), Centro Metropolitano, Conjunto A, lote 01, Ceilandia, Brasilia CEP, Brasília 72220-900, DF, Brazil; sabrinalunara@gmail.com (S.L.S.P.); acarolina.olive@gmail.com (A.C.A.d.O.F.); ricelle@unb.br (A.R.M.R.); cdssilva@gmail.com (C.M.d.S.S.); belbiomedica@gmail.com (I.C.R.d.S.)

**Keywords:** tetracycline, sulfonamide, *Salmonella*, antibiotic resistance

## Abstract

Tetracyclines and sulfonamides are broad-spectrum antibacterial agents which have been used to treat bacterial infections for over half a century. The widespread use of tetracyclines and sulfonamides led to the emergence of resistance in a diverse group of bacteria. This resistance can be studied by searching for resistance genes present in the bacteria responsible for different resistance mechanisms. *Salmonella* is one of the leading bacteria causing foodborne diseases worldwide, and its resistance to tetracyclines and sulfonamides has been widely reported. The literature review searched the Virtual Health Library for articles with specific data in the studied samples: the resistance genes found, the primers used in PCR, and the thermocycler conditions. The results revealed that *Salmonella* presented high rates of resistance to tetracycline and sulfonamide, and the most frequent samples used to isolate *Salmonella* were poultry and pork. The tetracycline resistance genes most frequently detected from *Salmonella* spp. were *tetA* followed by *tetB*. The gene *sul1* followed by *sul2* were the most frequently sulfonamide resistance genes present in *Salmonella*. These genes are associated with plasmids, transposons, or both, and are often conjugative, highlighting the transference potential of these genes to other bacteria, environments, animals, and humans.

## 1. Introduction

Tetracyclines are broad-spectrum antibacterial agents, which show activity against most Gram-positive and Gram-negative bacteria, both anaerobic and aerobic. The tetracyclines mode of action is well established; they inhibit bacterial protein synthesis by avoiding the association between RNA molecules and the 30S subunit of the bacterial ribosome, thus preventing the addition of amino acids and, consequently, protein synthesis [1,2,3,4,5,6]. 

Sulfonamides are synthetic antibacterial drugs presenting a para-amino benzoic acid (PABA) structure and containing a sulfonamide group linked to an aromatic group that competitively inhibits the enzyme dihydropteroate synthase (DHPS). DHPS participates in folate synthesis, an essential mechanism for bacterial DNA and RNA synthesis, using PABA as a substrate, and this competitive inhibition of DHPS by sulfonamides inhibits bacterial growth [7,8,9,10]. Consequently, these drugs have activity against a broad spectrum of bacteria, being able to inhibit both Gram-negative and Gram-positive bacteria that do not possess mechanisms to overcome the inhibition effects of DHPS [11]. 

Sulfonamides were the first drugs to be used in veterinary medicine in therapeutic doses [12,13]. Their excessive usage imposed widespread selective pressures on bacteria, as seen by the high prevalence rates of sulfonamide resistance observed in mainly Gram-negative bacteria isolated from animals and humans all over the world in the past decade [14,15,16,17]. Another concern is the accumulation of sulfonamides as environmental contaminants. Sulfonamides were a high priority of veterinary medicines, due to their high potential to reach the environment [18,19]. Sulfonamides are excreted after consumption and consequently, can be found at high concentrations in livestock wastewaters [20,21,22]. The accumulation of sulfonamides as environmental contaminants is potentiated by their resistance to degradation during conventional wastewater treatments [23]. In addition to the direct environmental adverse impacts, high sulfonamide concentrations increase the risks of food chain contamination [11]. 

Since the introduction of tetracyclines in 1950, their combination of broad-spectrum activity and low toxicity has led to their intensive use in human and animal infections therapy, and they have also been used for nearly as long to promote growth in food animal production systems [1]. The growth-promoting properties of tetracyclines were first described in 1949 for chickens, and farmers widely used them in animal husbandry thanks to improvement of the growth rate to feed intake ratio [12,13]. This extensive use favored the emergence of tetracycline resistance in a diverse group of bacteria and caused restrictions on the clinical utility of these compounds [2,3].

Tetracycline resistance in most bacteria is due to the acquisition of mobile genetic elements, ribosomal binding site mutations and chromosomal mutations leading to increased expression of intrinsic resistance mechanisms. Three principal tetracycline resistance mechanisms are efflux pumps, ribosomal protection, and enzymatic inactivation of tetracyclines drugs [1,3,24,25]. Several different *tet* genes have been described as conferring resistance to tetracyclines in bacteria. The most frequent types of *tet* genes belong to classes A, B, C, D and G [26], and these genes are responsible for encoding tetracycline efflux pumps [4,5,27,28]. Recent articles show that *Salmonella* spp. resistance to tetracycline is frequently found in analyzed samples, and this resistance is due mainly to the presence of *tet* genes in these bacteria. The *tetA*, *tetB*, *tetC* and *tetD* genes were detected on different *S. enterica* bacteria serotypes, including Typhimurium, Enteritidis, Hadar, Saintpaul and Choleraesuis [25,28,29,30].

Resistance to sulfonamides in Gram-negative bacteria is associated with the presence of *sul* genes that encode dihydropteroate synthase in a form that the drug cannot inhibit. There are four *sul* genes (*sul1*, *sul2*, *sul3* and *sul4*) that encode resistance to sulfonamides [7,10]. The *sul1* and *sul2* genes have previously been identified in *Enterobacteriaceae*, particularly *Escherichia* and *Salmonella* [10]. In 2003, Perreten and Boerlin [31] reported the *sul3* gene, detected in *Escherichia coli* isolated from pigs in Switzerland. In 2017, Razavi et al. [32] described the *sul4* gene, which provided clinical resistance in *Enterobacteriaceae*. *Sul* genes can be transferred between bacteria via integrons, transposons or plasmids [10]. According to Guerra et al. [33] the *sul3* gene can be detected in *Salmonella* spp. strains of different origins and serotypes on various large plasmids. However, dissemination of *sul1* and *sul2* genes among *Salmonella* spp. is reported more often than the *sul3* gene [7].

*Salmonella* is one of the most common bacteria that causes foodborne diseases worldwide [34]. The latest Brazilian foodborne disease national survey [35] reveals that, in the last nine years, *Salmonella* spp. was the second most common etiological agent identified in foodborne disease outbreaks in Brazil. Hoffmann et al. [36] reported that *Salmonella* causes more than one million diseases in the United States per year. Reports from the European Union in 2019 showed 87,923 confirmed cases of salmonellosis in humans, measuring up to 17.9% of foodborne outbreaks that year, with an observed overall high level of resistance to ampicillin, tetracyclines, and sulfonamides [37].

Some studies have shown that *Salmonella* has a higher percentage of tetracycline [38,39,40,41,42] and sulfonamide [7,14,16,21,43] resistance. There is a growing concern about the overall increase in bacterial resistance to antibiotics. Several studies have documented the transfer of antibiotic-resistant bacteria from animals to the human population, posing a serious threat to public health [43,44]. In this context, a literature review on the presence of tetracycline and sulfonamide resistance genes in *Salmonella* spp. was performed.

## 2. Materials and Methods 

### 2.1. Search Strategy

The bibliographic search was conducted through the Virtual Health Library (VHL), a portal where bibliographic reference databases and full texts are available to search for physical and digital books, booklets, manuals, magazines, and legislation, among other services. VHL also accesses international databases such as Medline and Lilacs, among others. Publications relating antimicrobial resistance genes for *Salmonella* spp. were screened using the following terms: “tetracycline resistance genes”, “sulfonamide resistance genes” and “*Salmonella*”. The retrieved publications were selected to be studied. 

### 2.2. Filters, Inclusion and Exclusion Criteria

According to the research interest, the terms were searched in the database from 2009 to 2019. The inclusion criteria were as follows: (1) the type of sample studied must have been reported; (2) the resistance genes sought; (3) the primers used in the polymerase chain reaction (PCR); and (4) thermocycler and PCR conditions. Studies were excluded if: (1) they had sought the resistance gene but did not present the primer sequence used in PCR; (2) the resistance gene was not towards tetracycline or sulfonamide; and (3) they did not have the thermocycler conditions used in PCR.

### 2.3. Data Extraction

Data were extracted from eligible studies according to the research criteria. For each study, the following characteristics were collected: the authors, the title of the study, the year of publication, the type of sample studied, the sample size, the resistance gene, the primers sequence of the genes, the thermocycler and PCR conditions, as well as the results.

## 3. Results and Discussion

Prevalence of tetracycline and sulfonamide resistant *Salmonella* spp. strains and distribution of tetracycline and sulfonamide resistance genes.

The search for articles associated with tetracycline and/or sulfonamide resistance genes to *Salmonella* spp. resulted in 25 studies that met the inclusion criteria (presented tetracycline and/or sulfonamide resistance genes, presented the primer sequence used in PCR and specified the thermocycler conditions used in PCR). Of the 25 studies, 6 searched for *tet* genes, 3 searched for *sul* genes, and 16 searched for both *tet* and *sul* genes. The general characteristics of the studies included in this review are summarized in Table 1. 

The percentage of tetracycline-resistant *Salmonella* spp. strains in relation to the total of *Salmonella* strains isolated in the studies varied from 25 to 100% (average of tetracycline-resistant isolates = 71.1%) (Table 2). Similarly, Mąka et al. [28] reported tetracycline resistance frequencies among *Salmonella* spp. strains isolated from various meats (pork, chicken, turkey, beef, and fish) were often 50.0% or higher (50–76%) in Brazil, Canada, Iran, India, Turkey, UK and Vietnam. A high frequency of *Salmonella* bacteria showed resistance to tetracycline (62–69%) in some studies [60,61,62].

Romero-Barrios et al. [63] isolated 1495 *Salmonella* strains in raw chicken products processed in slaughterhouses inspected by the Canadian federal government and sold at retail, and of these 642 (42.9%) strains showed resistance to tetracycline. Lopes et al. [52] isolated a total of 225 *Salmonella* strains from feed, pigs, and carcasses in Brazil and resistance was found most frequently to tetracycline (54.5%). Wang et al. [64] analyzed a total of 11.447 isolates of *S.* Typhimurium recovered from humans (n = 6381), animals (n = 2940), and retail meats (n = 2126), and tetracycline resistance was around 70% for *Salmonella* strains isolated from animals and meats, and around 40% for strains of human origin.

For sulfonamide, the percentage of resistant isolates in relation to the total of *Salmonella* strains in the studies varied from 5.2 to 100% (average of sulfonamide-resistant isolates = 57.4%) (Table 2). Other studies also reported high sulfonamide resistance in *Salmonella* strains [65,66,67,68,69]. Xu et al. [65] showed high *Salmonella* resistance to sulfonamide (73.0%) in the results for antimicrobial resistance profiles of strains isolated from chicken in China. Moe et al. [66] studied the antimicrobial resistance of *Salmonella* isolated from chicken carcasses in Myanmar and the isolates were most frequently resistant to trimethoprim-sulfamethoxazole (70.3%) and tetracycline (54.3%). 

Sodagari et al. [68] studied the antimicrobial resistance of *Salmonella* serotypes isolated from retail chicken meat in Iran and found high antimicrobial resistance rates were against tetracycline (81%) and sulfamethoxazole-trimethoprim (61.2%). Zeng et al. [69] determined the antimicrobial resistance of *Salmonella* in pork, chicken, and duck from retail markets in China, and the highest resistance was to trimethoprim–sulfamethoxazole (94.5%), followed by tetracycline (55.4%). 

Voss-Rech et al. [70] conducted a meta-analysis to assess the profile and temporal evolution of the antimicrobial resistance of nontyphoidal *Salmonella* isolated from poultry and humans in Brazil from 1995 to 2014. In the nontyphoidal isolates of poultry origin, the highest levels of antimicrobial resistance were verified for sulfonamides (44.3%), nalidixic acid (42.5%), and tetracycline (35.5%). In the human-origin isolates, the resistance occurred mainly for sulfonamides (46.4%), tetracycline (36.9%), and ampicillin (23.6%). Vaez et al. [71] also conducted a meta-analysis to determine the antimicrobial resistance profiles of *Salmonella* serotypes isolated from animals in Iran and isolates were mostly resistant against nalidixic acid (67%), then tetracycline (66.9%), followed by trimethoprim/sulfamethoxazole (41.6%).

**Figure 1:**
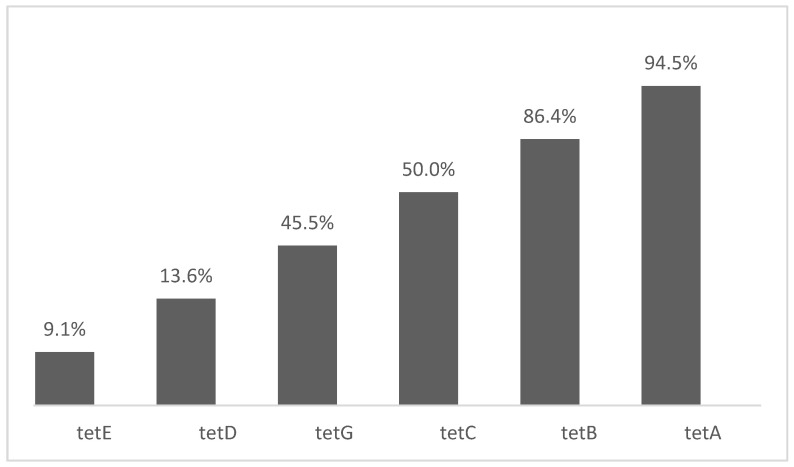
Percentage of studies that searched for tetracycline resistance genes.

The most searched tetracycline-resistance genes were: *tetA* with 21 studies (94.5%), *tetB* with 19 studies (86.4%), *tetC* with 11 studies (50.0%) and *tetG* with 10 studies (45.5%), while the least searched genes were *tetD* with 3 studies (13.6%) and *tetE* with 2 studies (9.1%) (Figure 1). The *tetA* gene was found in all 21 studies that searched for this gene, and its presence in *Salmonella* spp. strains varied from 8.0 to 87.5% (average of *tetA* gene in isolates = 47.7%). The *tetB* gene was found in 12 studies and its presence in *Salmonella* spp. strains varied from 0 to 75.0% (average of *tetB* gene in isolates = 28.3%). The *tetC* gene was present in 6 studies and its presence in *Salmonella* spp. strains varied from 0 to 86.6% (average of *tetC* gene in isolates = 19.9%). The *tetG* gene was found in 9 studies and its presence in *Salmonella* spp. strains varied from 0 to 26.0% (average of *tetG* gene in isolates = 8.4%). The *tetE* and *tetD* genes were not present in *Salmonella* spp. isolates (Table 3). 

Zhang et al. [72] reported that among 105 tetracycline-resistant *Salmonella*, *tetA* gene was most frequently detected (80.9%), and only 4.8% of isolates harbored *tetB* gene. The authors [73] reported that *tetA* and *tetB* genes are widely detected in fecal coliforms from rivers and animal sources. Matielo et al. [73] determined the antimicrobial resistance in *Salmonella enterica* strains isolated from Brazilian poultry production, and the genes *tetA*, *tetB* and *tetC* were detected in 60%, 5% and 5% of these isolates, respectively. Sanchez-Maldonado et al. [74] searched the antimicrobial resistance of *Salmonella* isolated from two pork processing plants in Canada, and the most prevalent genes were *tetB*, found in 21.3% of isolates and *tetA*, found in 12.6% of isolates.

According to Roberts and Schwarz [25], the *tetB* gene is specific for Gram-negative aerobic and facultative anaerobic bacteria, being present in 33 Gram-negative genera. If other aerobic and facultative anaerobic Gram-negative genes are of interest, the *tetA* gene is the next most common, being present in 23 Gram-negative genera. The *tet* genes are the most regularly found in *Enterobacteriaceae* [61]. The most common tetracycline resistance mechanism is antibiotic efflux pumps, in which *tet* genes encode the membrane-associated efflux proteins, which exchange a proton for a tetracycline-cation complex against a concentration gradient, exporting the drug to outside bacterial cells. These genes are generally associated with plasmids, transposons, or both and are often conjugative [2,3,28]. 

*Tet* genes belong to classes A, B, C, D and G are placed in the same group due to amino acid sequence similarity. The tetracycline resistance proteins in this group have from 41% to 78% amino acid identity [75]. Efflux of tetracyclines predominantly occurs via proteins that are members of the major facilitator superfamily group of integral membrane transporters. These efflux pumps are integral membrane proteins that span the lipid bilayer of the inner cell membrane. Based on homology to other known transporters, the membrane-spanning regions of the protein are predicted to be helical. The structure–function predicts a water-filled channel surrounded by six transmembrane helices. The tetracycline is predicted to pass through this channel and is exchanged for H^+^. It is this vectorial flow of protons through the channel, down the pH gradient, which provides the energy required to pump the antibiotic from the cell [76].

**Figure 2:**
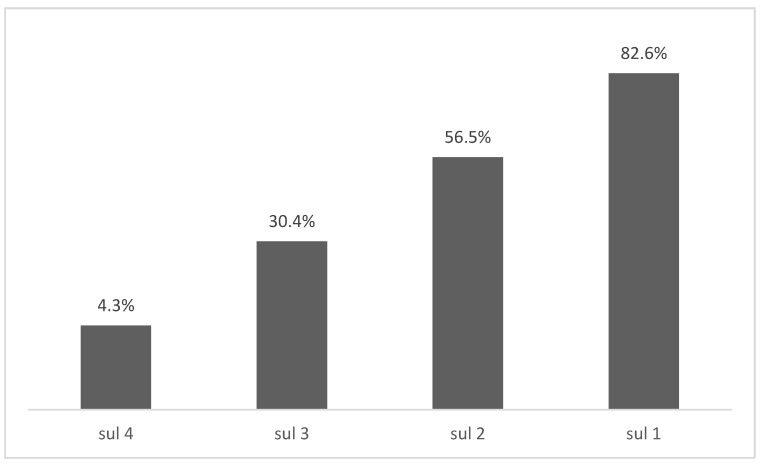
Percentage of studies that searched for sulfonamide resistance genes.

The most searched sulfonamide-resistance genes were: *sul1* with 19 studies (82.6%), *sul2* with 13 studies (56.5%), while the least searched genes were *sul3* with 7 studies (30.4%), and *sul4* with 1 study (4.3%) (Figure 2). The *sul1* gene was found in 18 of 19 studies that searched for this gene, and its presence in *Salmonella* spp. strains varied from 0 to 89.7% (average of *sul1* gene in isolates = 45.6%). The *sul2* gene was found in 12 studies and its presence in *Salmonella* spp. strains varied from 0 to 97.8% (average of *sul2* gene in isolates = 44.5%). The *sul3* gene was found in six studies and its presence in *Salmonella* spp. strains varied from 0 to 85.1% (average of *sul3* gene in isolates = 31.6%) (Table 3). 

Ma et al. [77] determined the antimicrobial resistance of *Salmonella* isolated from chickens and pigs on farms, abattoirs, and markets in Sichuan Province, China and among 74 strains carrying sulfonamides resistance gene, *sul1* was the most common (43.2%), followed by *sul2* (55.4%) and *sul3* (25.7%). Sanchez-Maldonado et al. [74] searched the antimicrobial resistance of *Salmonella* isolated from two pork processing plants in Alberta, Canada, and the most prevalent genes among those screened were *sul2*, found in 21.3% of isolates and *sul1*, found 18.1% of isolates. Zhu et al. [59] reported that the presence of the genes *sul1* and *sul2* was equal in *Salmonella* strains isolated from pork meat resistant to trimethoprim/sulfamethoxazole in China. 

Zhu et al. [43] reported that among 91 sulfonamide-resistant isolates, 97.8% (n = 89) harbored at least one of the genes studied (*sul1*, *sul2* or *sul3*). The *sul2* gene had the highest occurrence (97.8%, n = 89) compared to the *sul1* and *sul3* genes (both with 50.5%, n = 46). According to Mąka et al. [7] dissemination of *sul1* and *sul2* genes among *Salmonella* spp. is reported more often than *sul3* gene. Xu et al. [10] also reported that *sul1* and *sul2* genes are often found at roughly the same frequency among sulfonamide resistant Gram-negative isolates. According to Machado et al. [78] the presence of *sul* genes continues to be reported in surveys of environmental bacteria with *sul2* dominating but closely followed by *sul1*, and *sul3* is still rarer.

The *sul* genes are found in plasmids and are associated with ubiquitous and long-known sulfonamide resistance Gram-negative bacteria [10]. The *sul1* gene is typically found in class 1 integrons and linked to other resistance genes, whereas *sul2* gene is usually associated with small multicopy plasmids or large transmissible multiresistance plasmids [8,19]. The *sul3* gene was identified in conjugative plasmids in *E. coli*, while the *sul4* gene was identified in a systematic prospection of class 1 integron genes in Indian river sediments [8].

According to Perreten and Boerlin [31] *sul1* and *sul2* from *E. coli* share 57% of DNA identity and *sul3* revealed amino acid identities of 50.4% overall to *sul2* from *Salmonella enterica* subsp. *enterica* plasmid, and 40.9% to *sul1* from *E. coli* plasmid. Based on amino acid homology and phenotype, *sul3* was considered a new sulfonamide-resistant DHPS. According to Razavi et al. [32] *sul4* was identified with 31–33% identity to known mobile sulfonamide resistance genes (*sul1*, *sul2* and *sul3*). Based on its ability to provide sulfonamide resistance, its mobile character, as demonstrated by its presence in integrons, and the homology to previously known sulfonamide resistance genes, the name *sul4* was proposed. Structural prediction of *sul1*, *sul2*, *sul3* and *sul4* indicates strong overall similarities. The structure of the genes contains the binding sites for 7,8-dihydropterin pyrophosphate (DHPP), para-aminobenzoic acid (PABA), and sulfonamide. After DHPP has bound deep in the structure, sulfonamide binds near the surface of the protein. Thus, sulfonamide binding is affected by changes near the surface of DHPS [32].

The genes *sul1*, *sul2*, *sul3* and *sul4* can spread among bacteria of the same or different species by conjugation or transformation, thereby disseminating resistance genes [10,19]. Some studies about sulfonamide resistant isolates where none of these *sul* genes are detected have appeared in the literature, but so far, no other plasmid sulfonamide resistance gene has been reported [78,79].

Deekshit et al. [80] found that the *tetA* gene in strains of *Salmonella* spp. isolated from seafood in India was located on a plasmid and this gene was identical to *tetA* detected in other bacterial species including *Escherichia coli* and *Vibrio cholerae*. According to Vital et al. [41], large conjugative resistance plasmids have been detected in *Salmonella* food isolates from several countries. Conjugative plasmids can transfer several resistance genes between different bacterial species, and the presence of multiple antibiotic resistance genes facilitates their host survival despite intense antibiotic selection [25].

Selected *tet* genes are part of multiresistance elements, such as the integrative and mobilizable *Salmonella* genomic island 1. The majority of the tetracycline-resistance efflux genes have been linked to other antibiotic-resistance genes. These *tet* genes have been identified in environmental, animal and aquaculture-associated bacteria [81]. Hsu et al. [48] reported that high rates of bacterial resistance to antibiotics such as tetracycline are associated with the intensive use of these drugs in veterinary medicine. Hence, the emergence of resistant bacteria in the food chain has been a cause of great concern, even with the decline of tetracyclines use in clinical treatment [82,83].

Adesiji et al. [84] detected *tet*-resistant genes in *tet*-susceptible *Salmonella* isolates. The results show that some antimicrobial-resistant genes are silent in bacteria in vitro and indicate that these silent genes can turn on in vivo under selective antibiotic pressure or spread to other bacteria. These results reinforce the importance of determining *tet* and *sul* genes in addition to antimicrobial susceptibility tests. Wang et al. [85] also reported some silent or unexpressed *sul1* and *sul3* genes detected in the isolates of soils, which could be horizontally transferred or expressed under other conditions.

Table 4 presents the primer sequences and PCR conditions used to amplify resistance genes in the studies. The primer sequences used to amplify tetracycline and sulfonamide resistance genes in the studies were a vital inclusion criterion, as designing appropriate primers is essential to a successful PCR experiment outcome [86].

The target specificity is a critical primer property, and, ideally, a primer pair should only amplify the intended target. Several software tools have been developed to aid the primer design process. The Primer3 program is widely used in designs of the primers, however, it does not analyze the target of the primers specificity, so the user will need additional tools such as the software Primer-BLAST to test for specificity. This software ensures a complete primer-target alignment while being sensitive enough to detect a significant number of primer-target mismatches. Primer-BLAST software can also help design new target-specific primers in one step and check pre-existing specificity of the primers [87]. 

Another essential factor for the success of the experiment is the optimization of the conditions of the PCR. The choice of the correct thermal cycling conditions is vital to obtain better results in the research and replication of the method. In addition to bringing efficient results and reducing the attempts of the researcher, the optimization of PCR conditions also avoids some common problems, such as the amplifying of non-specific products or the absence of a product in the result [88].

The most frequent samples used in studies to isolate *Salmonella* spp. strains were: 13 samples from poultry-origin (52.0%), followed by 11 samples from swine-origin (44.0%) and 7 samples from bovine-origin (28.0%); while 4 studies used human samples, 2 studies used goat samples, 2 studies used water samples, 1 study used hen eggs, and another study used fresh vegetable samples (Table 5).

Salmonellosis is a significant zoonosis worldwide and is widespread in animals [89,90]. The present review found that the most frequent *Salmonella* isolates were from poultry and pork meat samples. Chicken meat is a widely consumed product worldwide, and different studies register contamination by *Salmonella* in this type of food [27,42,43]. Ren et al. [91] reported that the high contamination rates in the supply chain show that chicken products are an important vector of *S. enterica*. Previous studies have shown that the continuous circulation of *S. enterica* in the broiler supply system poses a potential risk of spreading *Salmonella* to humans [91,92,93,94,95].

*Salmonella* contamination in poultry and pigs is often asymptomatic and rarely causes less severe and transient diarrhea. Consumption of contaminated chicken and pork predisposes humans to *Salmonella* infection [42,43,96]. The presence of *Salmonella* in cattle in some studies [38,40,55] and the possibility of cross-contamination of the carcass in the slaughter of these animals may pose a risk to food safety in the consumption of this type of food [97].

*Salmonella* ssp. is an etiologic agent often cited as causing foodborne diseases [98,99]. In most cases, salmonellosis is caused by contaminated food products, particularly of animal origins such as poultry, eggs, beef, and pork [44]. The genetic constitution of these bacteria allows them to adapt to various environments and animals, including mammalian and non-mammalian hosts, making them widespread worldwide [82].

The abusive use of tetracycline and sulfonamides associated with the presence of *Salmonella* in different food sources has promoted the rise of resistant strains [42,81,99]. In Brazil, despite the ban on the use of antibiotics as performance enhancers in poultry production [100], tetracyclines have already been widely used as growth promoters. The presence of resistance genes found in this review suggests a remarkable ability of *Salmonella* spp. to survive in environments where antimicrobial agents are broadly used [42].

There is further concern regarding the release of these substances into the environment through hospital and industrial effluents, domestic sewage, and the disposal of expired drugs. Additionally, any resistance in potentially virulent strains of humans and animals can quickly spread, making their circulation in the environment more frequent [101,102,103,104,105].

## 4. Conclusions

The results obtained in this study revealed that the tetracycline resistance genes most frequently isolated from *Salmonella* spp. were *tetA* and *tetB*. The genes *sul1* and *sul2* were the most frequently sulfonamide-resistant genes present in *Salmonella*. The chicken and pork samples presented the most significant number of these resistance genes. The intensive use of tetracycline and sulfonamides antibiotics in the production chain of these foods must have resulted in the development of this resistance. Bacterial resistance represents a significant public health concern, as there is a possibility of transferring resistance genes between humans, animals, and the environment.

## Figures and Tables

**Table 1:** Summary of studies with tetracycline and sulfonamide resistance genes in *Salmonella* spp.

Studies	Authors	Title	Year	Genes Searched	Reference
1	Aslam et al.	Phenotypic and genetic characterization of antimicrobial resistance in *Salmonella* serovars isolated from retail meats in Alberta, Canada	2012	*tetA*, *tetB*, *tetC*, *sul1*, *sul2*, *sul3*	[45]
2	Dahshan et al.	Characterization of antibiotic resistance and the emergence of AmpC-producing *Salmonella infantis* from pigs	2010	*tetA*, *tetB*, *tetG*, *sul1*	[46]
3	Deng et al.	Antibiotic resistance in *Salmonella* from retail foods of animal origin and its association with disinfectant and heavy metal resistance	2017	*tetA*, *tetB*, *tetC*, *tetG*, *sul1*, *sul2*, *sul3*	[38]
4	Dessie et al.	Characterization of integrons and their cassettes in *Escherichia coli* and *Salmonella* isolates from poultry in Korea	2013	*tetA*, *tetB*, *tetC*, *tetD*, *tetE*, *tetG*, *sul1*, *sul2*	[27]
5	El-Sharkawy et al.	Epidemiological, molecular characterization and antibiotic resistance of *Salmonella enterica* serovars isolated from chicken farms in Egypt	2017	*tetA*, *tetB*, *tetC*, *sul1*, *sul2*, *sul3*	[47]
6	Hsu et al.	Antibiotic resistance pattern and gene expression of non-typhoid *Salmonella* in river sheds	2014	*tetA*, *tetB*, *sul1*	[48]
7	Igbinosa	Prevalence and detection of antibiotic-resistant determinant in *Salmonella* isolated from food-producing animals	2014	*tetC*	[44]
8	Iwu et al.	Multidrug-resistant *Salmonella* isolates from swine in the Eastern Cape Province, South Africa	2016	*tetA*	[39]
9	Khoshbakht et al.	Tetracycline resistance genes in *Salmonella enterica* serovars with animal and human origin	2018	*tetA*, *tetB*, *tetC*, *tetG*	[49]
10	Kozak et al.	Distribution of sulfonamide resistance genes in *Escherichia coli* and *Salmonella* isolates from swine and chickens at Abattoirs in Ontario and Québec, Canada	2009	*sul1*, *sul2*, *sul3*	[50]
11	Lapierre et al.	Comparison of integron-linked antibiotic resistance genes in strains of *Salmonella* spp. isolated from swine in Chile in 2005 and 2008	2010	*tetA*, *tetB*, *tetG*	[51]
12	Lopes et al.	Resistance phenotypes and genotypes of *Salmonella enterica* subsp. *enterica* isolates from feed, pigs, and carcasses in Brazil	2015	*tetA*, *tetB*, *sul1*, *sul2*, *sul3*	[52]
13	Maka et al.	Resistance to sulfonamides and dissemination of *sul* genes among *Salmonella* spp. isolated from food in Poland	2015	*sul1*, *sul2*, *sul3*	[7]
14	Marquéz et al.	Biocide tolerance and antibiotic resistance in *Salmonella* isolates from hen eggshells	2017	*tetA*, *tetB*, *tetC*, *tetD*, *tetE*, *tetG*, *sul1*	[53]
15	Mthembu et al.	Molecular detection of multidrug-resistant *Salmonella* isolated from livestock production systems in South Africa	2019	*tetA*, *tetC*, *sul2*	[54]
16	Sadiq et al.	Antibacterial activities and possible modes of action of *Acacia nilotica* (L.) Del. against multidrug-resistant *Escherichia coli* and *Salmonella*	2017	*tetA*, *tetB*	[40]
17	Soyer et al.	Antimicrobial drug resistance patterns among cattle-and human-associated *Salmonella* strains	2013	*tetA*, *tetB*, *tetG*, *sul1*, *sul2*	[55]
18	Tajbakhsh et al.	Antimicrobial resistance in *Salmonella* spp. recovered from patients admitted to six different hospitals in Tehran, Iran from 2007 to 2008	2012	*tetA*, *tetB*, *tetC*, *tetD*, *tetG*, *sul1*	[56]
19	Thai et al.	Antimicrobial resistance in *Salmonella serovars* isolated from meat shops at markets in North Vietnam.	2012	*tetA*, *tetB*, *tetG*, *sul1*	[57]
20	Vital et al.	Antimicrobial resistance in *Escherichia coli* and *Salmonella* spp. isolates from fresh produce and the impact to food safety.	2017	*tetA*, *tetB*, *tetC*	[41]
21	Vuthy et al.	Antibiotic susceptibility and molecular characterization of resistance genes among *Escherichia coli* and among *Salmonella* subsp. in chicken food chains.	2017	*tetA*, *tetB*, *sul1*, *sul2*	[58]
22	Xu et al.	Development and evaluation of a Luminex xTAG assay for sulfonamide resistance genes in *Escherichia coli* and *Salmonella* isolates	2019	*sul1*, *sul2*, *sul3*, *sul4*	[10]
23	Zhu et al.	Antimicrobial resistance and resistance genes in *Salmonella* strains isolated from broiler chickens along the slaughtering process in China	2017	*tetA*, *tetB*, *tetC*, *tetG*, *sul1*, *sul2*, *sul3*	[43]
24	Zhu et al.	Surveillance study of the prevalence and antimicrobial resistance of *Salmonella* in pork from open markets in Xuzhou, China	2019	*tetA*, *tetB*, *sul1*, *sul2*	[59]
25	Zishiri et al.	Prevalence of virulence and antimicrobial resistance genes in *Salmonella* spp. isolated from commercial chickens and human clinical isolates from South Africa and Brazil	2016	*tetA*, *tetB*, *sul1*, *sul2*	[42]

**Table 2:** Prevalence of tetracycline and sulfonamide resistance in relation to the total number of *Salmonella* isolates.

Studies	No. of *Salmonella* Isolates	Tetracycline-Resistant Isolates n (%)	Isolates with *tet* Genes n (%)	Sulfonamide-Resistant Isolates n (%)	Isolates with *sul* Genes n (%)
Aslam et al. 2012 [45]	110	54 (49.0%)	45 (40.9%)	9 (8.0%)	9 (8.0%)
Dahshan et al. 2010 [46]	44	44 (100%)	10 (22.7%)	44 (100%)	8 (18.2%)
Deng et al. 2017 [38]	152	123 (80.9%)	123 (80.9%)	98 (64.5%)	60 (39.5%)
Dessie et al. 2013 [27]	33	23 (69.7%)	8 (24.2%)	31 (93.9%)	26 (78.8%)
El-Sharkawy et al. 2017 [47]	67	61 (91.0%)	58 (86.6%)	3 (5.2%)	58 (86.6%)
Hsu et al. 2014 [48]	54	18 (33.3%)	14 (26.0%)	20 (37.0%)	16 (29.6%)
Igbinosa 2015 [44]	150	73 (48.7%)	0	99 (66.0%)	*
Iwu et al. 2016 [39]	48	48 (100%)	30 (61.0%)	36 (75.0%)	*
Khoshbakht et al. 2018 [49]	60	60 (100%)	6 (10.0%)	*	*
Kozak et al. 2009 [50]	234	*	*	*	210 (89.7%)
Lapierre et al. 2010 [51]	69	65 (94.2%)	49 (71.0%)	19 (27.5%)	*
Lopes et al. 2015 [52]	225	122 (54.5%)	73 (32.5%)	89 (39.6%)	65 (28.9%)
Maka et al. 2015 [7]	84	*	*	84 (100%)	76 (90.5%)
Marquéz et al. 2017 [53]	39	19 (47.6%)	6 (14.3%)	15 (38.1%)	4 (9.5%)
Mthembu et al. 2019 [54]	106	67 (63.0%)	25 (26.0%)	41 (38.0%)	22 (21.0%)
Sadiq et al. 2017 [40]	4	3 (75.0%)	3 (75.0%)	*	*
Soyer et al. 2013 [55]	336	296 (88.0%)	44 (13.1%)	282 (84.0%)	49 (14.6%)
Tajbakhsh et al. 2012 [56]	71	18 (25.0%)	34 (48.0%)	21 (30.0%)	23 (32.0%)
Thai et al. 2012 [57]	97	47 (48.5%)	40 (41.2%)	55 (56.7%)	52 (53.6%)
Vital et al. 2017 [41]	24	16 (66.7%)	21 (87.5%)	*	*
Vuthy et al. 2017 [58]	181	157 (86.7%)	117 (64.6%)	156 (86.2%)	78 (43.1%)
Xu et al. 2019 [10]	18	*	*	13 (72.2%)	14 (77.8%)
Zhu et al. 2017 [43]	189	98 (51.9%)	84 (44.4%)	91 (48.1%)	89 (47.1%)
Zhu et al. 2019 [59]	155	143 (92.0%)	32 (20.6%)	81 (52.2%)	29 (18.7%)
Zishiri et al. 2016 [42]	146	136 (93.0%)	128 (87.7%)	123 (84.0%)	125 (85.6%)

* Antimicrobials were not tested, or genes were not searched in the study.

**Table 3:** Distribution of tetracycline and sulfonamide resistance genes in relation to *Salmonella* isolates with.

Studies	*Salmonella* Isolates (n)	*tet* and *sul* Genes in *Salmonella* Isolates n (%)									
		*tetA*	*tetB*	*tetC*	*tetD*	*tetE*	*tetG*	*sul1*	*sul2*	*sul3*	*sul4*
Aslam et al. 2012 [45]	45 *tet* 9 *sul*	31 (68.7%)	14 (31.2%)	0%	*	*	*	5 (55.6%)	3 (33.3%)	1 (11.2%)	*
Dahshan et al. 2010 [46]	10 *tet* 10 *sul*	6 (60.0%)	2 (20.0%)	*	*	*	2 (20.0%)	8 (80.0%)	*	*	*
Deng et al. 2017 [38]	123 *tet* 60 *sul*	54 (44.7%)	11 (9.0%)	42 (34.1%)	*	*	27 (21.9%)	20 (33.3%)	20 (33.3%)	20 (33.3%)	*
Dessie et al. 2013 [27]	33 *tet* 33 *sul*	8 (24.2%)	0%	0%	0%	0%	0%	0%	26 (78.8%)	*	*
El-Sharkawy et al. 2017 [47]	67 *tet* 67 *sul*	55 (82.0%)	0%	58 (86.6%)	*	*	*	34 (50.7%)	0%	57 (85.1%)	*
Hsu et al. 2014 [48]	54 *tet* 54 *sul*	13 (24.1%)	1 (1.9%)	*	*	*	*	16 (29.6%)	*	*	*
Igbinosa 2015 [44]	73 *tet*	*	*	0%	*	*	*	*	*	*	*
Iwu et al. 2016 [39]	48 *tet*	30 (61.0%)	*	*	*	*	*	*	*	*	*
Khoshbakht et al. 2018 [49]	60 *tet*	6 (10.0%)	0%	3 (5.0%)	*	*	0%	*	*	*	*
Kozak et al. 2009 [50]	234 *sul*	*	*	*	*	*	*	180 (76.9%)	25 (10.7%)	5 (2.1%)	*
Lapierre et al. 2010 [51]	65 *tet*	10 (15.4%)	39 (60.0%)	*	*	*	0%	*	*	*	*
Lopes et al. 2015 [52]	91 *tet* 91 *sul*	61 (67.0%)	30 (32.9%)	*	*	*	*	47 (51.6%)	14 (15.4%)	11 (12.1%)	*
Maka et al. 2015 [7]	84 *sul*	*	*	*	*	*	*	37 (44.0%)	39 (46.4%)	0	*
Marquéz et al. 2017 [53]	39 *tet* 39 *sul*	4 (9.5%)	0%	2 (4.8%)	0%	0%	0%	4 (9.5%)	*	*	*
Mthembu et al. 2019 [54]	106 *tet* 106 *sul*	9 (8.0%)	*	19 (18.0%)	*	*	*	22 (21.0%)	*	*	*
Sadiq et al. 2017 [40]	4 *tet*	2 (50.0%)	3 (75.0%)	*	*	*	*	*	*	*	*
Soyer et al. 2013 [55]	48 *tet* 48 *sul*	36 (75.0%)	3 (6.3%)	*	*	*	5 (10.4%)	23 (47.9%)	26 (54.2%)	*	*
Tajbakhsh et al. 2012 [56]	71 *tet* 71 *sul*	20 (28.0%)	10 (14.0%)	0%	0%	*	4 (6.0%)	23 (32.0%)	*	*	*
Thai et al. 2012 [57]	50 *tet* 58 *sul*	37 (74.0%)	3 (6.0%)	*	*	*	13 (26.0%)	52 (89.7%)	*	*	*
Vital et al. 2017 [41]	24 *tet*	21 (87.5%)	0%	0%	*	*	*	*	*	*	*
Vuthy et al. 2017 [58]	157 *tet* 156 *sul*	117 (64.6%)	0%	*	*	*	*	39 (25.0%)	38 (24.3%)	*	*
Xu et al. 2019 [10]	18 *sul*	*	*	*	*	*	*	10 (55.6%)	13 (72.2%)	5 (27.8%)	1 (5.6%)
Zhu et al. 2017 [43]	98 *tet* 91 *sul*	23 (23.5%)	49 (50.0%)	70 (71.4%)	*	*	0%	43 (50.0%)	89 (97.8%)	43 (50.0%)	*
Zhu et al. 2019 [59]	29 *sul* 45 *tet*	32 (71.1%)	0%	*	*	*	*	18 (62.1%)	18 (62.1%)	*	*
Zishiri et al. 2016 [42]	146 *tet* 146 *sul*	79 (54.1%)	49 (33.6%)	*	*	*	*	76 (52.1%)	74 (50.7%)	*	*

* genes were not searched in the study.

**Table 4:** Primer sequences and PCR conditions used for the amplification of tetracycline and sulfonamide resistance genes.

Authors	Genes Searched	Primers	PCR Amplification Conditions
Aslam et al. [45]	*tetA*	F: GGCGGTCTTCTTCATCATGC R: CGGCAGGCAGAGCAAGTAGA	Initial denaturation at 94 °C for 15 min, followed by 30 cycles of denaturation at 94 °C for 1 min, annealing at 63 °C for 1 min, and extension at 72 °C for 1 min, with an additional extension at 72 °C for 10 min.
	*tetB*	F: CGCCCAGTGCTGTTGTTGTC R: CGCGTTGAGAAGCTGAGGTG	
	*tetC*	F: GCTGTAGGCATAGGCTTGGT R: GCCGGAAGCGAGAAGAATCA	
	*sul1*	F: CGGCGTGGGCTACCTGAACG R: GCCGATCGCGTGAAGTTCCG	Initial denaturation at 95 °C for 15 min, followed by 30 cycles of denaturation at 95 °C for 1 min, annealing at 66 °C for 1 min, and extension at 72 °C for 1 min, with an additional extension at 72 °C for 10 min.
	*sul2*	F: CGGCATCGTCAACATAACCT R: TGTGCGGATGAAGTCAGCTC	
	*sul3*	F: CAACGGAAGTGGGCGTTGTGGA R: GCTGCACCAATTCGCTGAACG	
Dahshan et al. [46]	*tetA*	F: GCTACATCCTGCTTGCCTTC R: CATAGATCGCCGTGAAGAGG	Annealing temperature: 64 °C
	*tetB*	F: TTGGTTAGGGGCAAGTTTTG R: GTAATGGGCCAATAACACCG	
	*tetG*	F: GCTCGGTGGTATCTCTGCTC R: AGCAACAGAATCGGGAACAC	Annealing temperature: 59 °C
	*sul1*	F: TCGGATCAGACGTCGTGG R: CCAGCCTGCAGTCCGCCT	Annealing temperature: 60 °C
Deng et al. [38]	*tetA*	F: CTCAGTATTCCAAGCCTTTG R: ACTCCCCTGAGCTTGAGGGG	30 cycles of denaturation at 94 °C for 1 min, annealing at 60 °C for 45 s, and extension at 72 °C for 90 s, with an additional extension at 72 °C for 5 min.
	*tetB*	F: CTAATCTAGACATCATTAATTCC R: TTTGAAGCTAAATCTTCTTTAT	
	*tetG*	F: AGTTTCAGGTGCGCAGC R: CCAATCGCCATGACTAAT	
	*sul1*	F: CATCATTTTCGGCATCGTC R: TCTTGCGGTTTCTTTCAGC	Initial denaturation at 94 °C for 5 min, followed by 35 cycles of denaturation at 94 °C for 50 s, annealing at 54 °C for 50 s, and extension at 72 °C for 1 min, with an additional extension at 72 °C for 10 min.
	*sul2*	F: AGATGTGATTGATTTGGGAGC R: TAGTTGTTTCTGGATTAGAGCCT	
	*sul3*	F: CTTCGATGAGAGCCGGCGGC R: GCAAGGCGGAAACCCGCGCC	
Dessie et al. [27]	*tetA*	F: GTAATTCTGAGCACTGTCGC R: CTGCCTGGACAACATTGCTT	Initial denaturation at 94 °C for 4 min, followed by 34 cycles of denaturation at 94 °C for 1 min, annealing at 43 °C for 2 min, and extension at 72 °C for 3 min, with an additional extension at 72 °C for 7 min.
	*tetB*	F: CTCAGTATTCCAAGCCTTTG R: ACTCCCCTGAGCTTGAGGGG	
	*tetC*	F: CCTCTTGCGGGATATCGTCC R: GGTTGAAGGCTCTCAAGGGC	
	*tetD*	F: GGATATCTCACCGCATCTGC R: CATCCATCCGGAAGTGATAGC	
	*tetE*	F: AAACCACATCCTCCATACGC R: AAATAGGCCACAACCGTCAG	
	*sul1*	F: CTTCGATGAGAGCCGGCGGC R: GCAAGGCGGAAACCCGCGCC	Initial denaturation at 94 °C for 5 min, followed by 30 cycles of denaturation at 94 °C for 15 s, annealing at 69 °C for 30 s, and extension at 72 °C for 1 min, with an additional extension at 72 °C for 7 min.
	*sul2*	F: CGGCATCGTCAACATAACC R: GTGTGCGGATGAAGTCAG	
El-Sharkawy et al. [47]	*tetA*	F: GCTACATCCTGCTTGCCTTC R: CATAGATCGCCGTGAAGAGG	Initial denaturation at 94 °C for 5 min, followed by 35 cycles of denaturation at 94 °C for 1 min, annealing at 55 °C for 2 min, and extension at 72 °C for 90 s.
	*tetB*	F: TTGGTTAGGGGCAAGTTTTG R: GTAATGGGCCAATAACACCG	Same conditions, with the specific annealing temperature: 53 °C
	*tetC*	F: CTTGAGAGCCTTCAACCCAG R: ATGGTCGTCATCTACCTGCC	Same conditions, with the specific annealing temperature: 56 °C
	*sul1*	F: TCACCGAGGACTCCTTCTTC R: AATATCGGGATAGAGCGCAG	Initial denaturation at 94 °C for 3 min, followed by 35 cycles of denaturation at 94 °C for 1 min, specific annealing temperature at 60 °C, and extension at 72 °C for 1 min, with an additional extension at 72 °C for 7 min.
	*sul2*	F: CGGTCCGGCATCCAGCAATCC R: CGAGAGCCACGACCGCGCC	Same conditions, with the specific annealing temperature: 64 °C
	*sul3*	F: GAGCAAGATTTTTGGAATCG R: CATCTGCAGCTAACCTAGGGCTTGGA	Same conditions, with the specific annealing temperature: 51 °C
Hsu et al. [48]	*tetA*	F: GCTACATCCTGCTTGCCTTC R: CATAGATCGCCGTGAAGAGG	Annealing temperature: 55 °C
	*tetB*	F: TTGGTTAGGGGCAAGTTTTG R: GTAATGGGCCAATAACACCG	
	*sul1*	F: TCGGATCAGACGTCGTGG R: CCAGCCTGCAGTCCGCCT	Annealing temperature: 60 °C
Igbinosa [44]	*tetC*	F: GGTTGAAGGCTCTCAAGGGC R: GGTTGAAGGCTCTCAAGGGC	Initial denaturation at 94 °C for 3 min, followed by 30 cycles of denaturation at 94 °C for 1 min, annealing at 65 °C for 1 min, and extension at 72 °C for 1 min, with an additional extension at 72 °C for 10 min.
Iwu et al. [39]	*tetA*	F: GGCCTCAATTTCCTGACG R: AAGCAGGATGTAGCCTGTGC	Initial denaturation at 94 °C for 5 min, followed by 35 cycles of denaturation at 94 °C for 1 min, annealing at 55 °C for 1 min, and extension at 72 °C for 1.5-min, with an additional extension at 72 °C for 5 min.
Khoshbakht et al. [49]	*tetA*	F: GCTACATCCTGCTTGCCTTC R: CATAGATCGCCGTGAAGAGG	Annealing temperature: 50 °C
	*tetB*	F: TTGGTTAGGGGCAAGTTTTG R: GTAATGGGCCAATAACACCG	
	*tetC*	F: CTTGAGAGCCTTCAACCCAG R: ATGGTCGTCATCTACCTGCC	Annealing temperature: 49 °C
	*tetG*	F: GCTCGGTGGTATCTCTGCTC R: AGCAACAGAATCGGGAACAC	
Kozak et al. [50]	*sul1*	F: CGGCGTGGGCTACCTGAACG R: GCCGATCGCGTGAAGTTCCG	Initial denaturation at 95 °C for 15 min, followed by 30 cycles of denaturation at 95 °C for 1 min, annealing at 66 °C for 1 min, and extension at 72 °C for 1 min, with an additional extension at 72 °C for 10 min.
	*sul2*	F: CGGCATCGTCAACATAACCT R: TGTGCGGATGAAGTCAGCTC	
	*sul3*	F: CAACGGAAGTGGGCGTTGTGGA R: GCTGCACCAATTCGCTGAACG	
Lapierre et al. [51]	*tetA*	F: GGTTCACTCGAACGACGTCA R: CTGTCCGACAAGTTGCATGA	Annealing temperature: 52 °C
	*tetB*	F: CTGGATTACTTATTGCTGGC R: CACCTTGCTGATGACTCTT	
	*tetG*	F: CCGGTCTTATGGGTGCTCTA R: GACTGGCTTCGTTCTTCTGG	Annealing temperature: 56 °C
Lopes et al. [52]	*tetA*	F: GTAATTCTGAGCACTGT R: CCTGGACAACATTGCTT	Initial denaturation at 94 °C for 4 min, followed by 34 cycles of denaturation at 94 °C for 1 min, annealing at 43 °C for 2 min, and extension at 72 °C for 3 min, with an additional extension at 72 °C for 7 min.
	*tetB*	F: ACGTTACTCGATGCCAT R: AGCACTTGTCTCCTGTT	
	*tetG*	F: CTGCTGATCGTGGGTCT R: TTGCGAATGGTCTGCGT	
	*sul1*	F: ATGGTGACGGTGTTCGGCATTCTGA R: CTAGGCATGATCTAACCCTCGGTCT	Initial denaturation at 94 °C for 2 min, followed by 30 cycles of denaturation at 94 °C for 1 min, annealing at 51 °C for 1 min, and extension at 72 °C for 1 min, with an additional extension at 72 °C for 7 min.
	*sul2*	F: ACAGTTTCTCCGATGGAGGCC R: CTCGTGTGTGCGGATGAAGTC	Same conditions, with the specific annealing temperature of 64 °C
	*sul3*	F: GAGCAAGATTTTTGGAATCG R: CATCTGCAGCTAACCTAGGGCTTTGGA	Same conditions, with the specific annealing temperature of 51 °C
Maka et al. [7]	*sul1*	F: CGGCGTGGGCTACCTGAACG R: GCCGATCGCGTGAAGTTCCG	Initial denaturation at 94 °C for 5 min, followed by 30 cycles of denaturation at 94 °C for 30 s, annealing at 68 °C for 25 s, and extension at 72 °C for 1 min, with an additional extension at 72 °C for 10 min.
	*sul2*	F: GCGCTCAAGGCAGATGGCAT R: GCGTTTGATACCGGCACCCGT	
	*sul3*	F: CAGATAAGGCAATTGAGCATGCTCTGC R: AGAATGATTTCCGTGACACTGCAATCATT	
Marquéz et al. [53]	*tetA*	F: GCTACATCCTGCTTGCCTTC R: CATAGATCGCCGTGAAGAGG	Initial denaturation at 94 °C for 5 min, followed by 35 cycles of denaturation at 94 °C for 1 min, annealing at 55 °C for 1 min, and extension at 72 °C for 1-5 min.
	*tetB*	F: TTGGTTAGGGGCAAGTTTTG R: GTAATGGGCCAATAACACCG	
	*tetC*	F: CTTGAGAGCCTTCAACCCAG R: ATGGTCGTCATCTACCTGCC	
	*tetD*	F: AAACCATTACGGCATTCTGC R: GACCGGATACACCATCCATC	
	*tetE*	F: AAACCACATCCTCCATACGC R: AAATAGGCCACAACCGTCAG	
	*tetG*	F: GCTCGGTGGTATCTCTGCTC R: AGCAACAGAATCGGGAACAC	
	*sul1*	F: CTTCGATGAGAGCCGGCGGC R: GCAAGGCGGAAACCCGCGCC	Annealing temperature: 65 °C for 30 s
Mthembu et al. [54]	*tetA*	F: GCTACATCCTGCTTGCCTTC R: CATAGATCGCCGTGAAGAGG	Initial denaturation at 95 °C for 3 min, followed by 30 cycles of denaturation at 95 °C for 30 s, annealing at 52 °C for 30 s, and extension at 72 °C for 1 min, with an additional extension at 72 °C for 8 min.
	*tetC*	F: CTTGAGAGCCTTCAACCCAG R: ATGGTCGTCATCTACCTGCC	Same conditions, with the specific annealing temperature: 42 °C
	*sul2*	F: CGGCATCGTCAACATAACC R: GTGTGCGGATGAAGTCAG	Same conditions, with the specific annealing temperature: 60 °C
Sadiq et al. [40]	*tetA*	F: GGTTCACTCGAACGACGTCA R: CTGTCCGACAAGTTGCATGA	Initial denaturation at 95 °C for 30 s, followed by 30 cycles of denaturation at 95 °C for 30 s, annealing at 61.1 °C for 30 s, and extension at 68 °C for 1 min, with an additional extension at 68 °C for 5 min.
	*tetB*	F: CCTCAGCTTCTCAACGCGTG R: GCACCTTGCTGATGACTCT	
Soyer et al. [55]	*tetA*	F: GCGCCTTTCCTTTGGGTTCT R: CCACCCGTTCCACGTTGTTA	
	*tetB*	F: CCCAGTGCTGTTGTTGTCAT R: CCACCACCAGCCAATAAAAT	
	*tetG*	F: AGCAGGTCGCTGGACACTAT R: CGCGGTGTTCCACTGAAAAC	Initial denaturation at 95 °C for 10 min, followed by 32 to 35 cycles of denaturation at 95 °C for 30 s, annealing at 55 °C for 1 min, and extension at 72 °C for 1 min, with an additional extension at 72 °C for 7 min.
	*sul1*	F: TCACCGAGGACTCCTTCTTC R: CAGTCCGCCTCAGCAATATC	
	*sul2*	F: CCTGTTTCGTCCGACACAGA R: GAAGCGCAGCCGCAATTCAT	
Tajbakhsh et al. [56]	*tetA*	F: GTAATTCTGAGCACTGTCGC R: CTGCCTGGACAACATTGCTT	Annealing temperature: 58 °C
	*tetB*	F: TTGGTTAGGGGCAAGTTTTG R: GTAATGGGCCAATAACACCG	Annealing temperature: 60 °C
	*tetC*	F: ATGGTCGTCATCTACCTGCC R: GGTTGAAGGCTCTCAAGGGC	Annealing temperature: 53 °C
	*tetD*	F: AAACCATTACGGCATTCTGC R: GACCGGATACACCATCCATC	Annealing temperature: 60 °C
	*tetG*	F: CAGCTTTCGGATTCTACGG R: GATTGGTGAGGCTCGTTAGC	
Thai et al. [57]	*tetA*	F: GCTACATCCTGCTTGCCT R: CATAGATCGCCGTGAAGA	Initial denaturation at 94 °C for 5 min, followed by 30 cycles of denaturation at 94 °C for 30 s, the corresponding temperature of each primer pair for 30 s, and extension at 72 °C for 1 min, with an additional extension at 72 °C for 5 min.
	*tetB*	F: TTGGTTAGGGGCAAGTTTTG R: GTAATGGGCCAATAACACCG	
	*tetG*	F: GCTCGGTGGTATCTCTGC R: AGCAACAGAATCGGGAAC	
	*sul1*	F: CTTCGATGAGAGCCGGCGGC R: GCAAGGCGGAAACCCGCGCC	
Vital et al. [41]	*tetA*	F: GTGAAACCCAACATACCCC R: GAAGGCAAGCAGGATGTAG	Initial denaturation at 94 °C for 5 min, followed by 30 cycles of denaturation at 94 °C for 30 s, annealing at 50º C for 30 s, and extension at 72 °C for 1 min, with an additional extension at 72 °C for 10 min.
	*tetB*	F: CCTTATCATGCCAGTCTTGC R: ACTGCCGTTTTTTCGCC	
	*tetC*	F: ACTTGGAGCCACTATCGAC R: CTACAATCCATGCCAACCC	
Vuthy et al. [58]	*tetA*	F: GCTACATCCTGCTTGCCTTC R: CATAGATCGCCGTGAAGAGG	Annealing temperature: 58 °C
	*tetB*	F: TTGGTTAGGGGCAAGTTTTG R: GTAATGGGCCAATAACACCG	
	*sul1*	F: GTGACGGTGTTCGGCATTCT R: TTTACAGGAAGGCCAACGGT	
	*sul2*	F: GGCAGATGTGATCGACCTCG R: ATGCCGGGATCAAGGACAAG	
Xu et al. [10]	*sul1*	F: CTAAACATACAAATACACATTTCA R: TGAAGTTCCGCCGCAAGGCTCG	Initial denaturation at 95 °C for 5 min, followed by 35 cycles of denaturation at 95 °C for 30 s, annealing at 58º C for 30 s, and extension at 72 °C for 15 s, with an additional extension at 72 °C for 8 min. Initial denaturation at 95 °C for 5 min, followed by 35 cycles of denaturation at 94 °C for 30 s, annealing at 63º C for 30 s, and extension at 72 °C for 90 s, with an additional extension at 72 °C for 5 min.
	*sul2*	F: TACTTAAACATACAAACTTACTCA R: TGCCAAACTCGTCGTTATGC	
	*sul3*	F: ATCTCAATTACAATAACACACAAA R: CGGGTATGGGCTTCTTTTTAG	
	*sul4*	F: TACTACTTCTATAACTCACTTAAA R: CGGACCTATTAAGATGGGAAA	
Zhu et al. [43]	*tetA* *tetB*	F: GTAATTCTGAGCACTGTCGC R: GAGACGCAATCGAATTCGG F: GAGACGCAATCGAATTCGG R: TTTAGTGGCTATTCTTCCTGCC	Initial denaturation at 95 °C for 10 min, followed by 35 cycles of denaturation at 94 °C for 45 s, annealing at 55-70º C for 50 s, and extension at 72 °C for 50 s, with an additional extension at 72 °C for 10 min.
	*tetC*	F: CTTGAGAGCCTTCAACCCAG R: ATGGTCGTCATCTACCTGCC	
	*tetG*	F: GCTCGGTGGTATCTCTGCTC R: AGCAACAGAATCGGGAACAC	
	*sul1*	F: CTTCGATGAGAGCCGGCGGC R: GCAAGGCGGAAACCCGCGCC	
	*sul2*	F: GCGCTCAAGGCAGATGGCATT R: GCGTTTGATACCGGCACCCGT	
	*sul3*	F: AGATGTGATTGATTTGGGAGC R: TAGTTGTTTCTGGATTAGAGCCT	
Zhu et al. [59]	*tetA*	F: TCGCTTGCCGCATTT R: CGCGTATAGCTTGCCG	Initial denaturation at 94 °C for 5 min, followed by 30 cycles of denaturation at 94 °C for 30 s, annealing at 55º C for 30 s, and extension at 72 °C for 1 min, with an additional extension at 72 °C for 6 min.
	*tetB*	F: GACACTCTATCATTGAT R: GACAATATTTAGCAACG	
	*sul1*	F: TGCAGGCTGGTGGTGGTTA R: CGCGTGGGTGCGGACGT	
	*sul2*	F: CATTCCCGTCTCGCTCGA R: GCGCGCAGAAAGGATTT	
Zishiri et al. [42]	*tetA*	F: GCTACATCCTGCTTGCCTT R: CATAGATCGCCGTGAAGAGG	Initial denaturation at 94 °C for 5 min, followed by 34 cycles of denaturation at 94 °C for 25 s, annealing at 55º C for 50 s, and extension at 72 °C for 50 s, with an additional extension at 72 °C for 5 min.
	*tetB*	F: TTGGTTAGGGGCAAGTTTTG R: GTAATGGGCCAATAACACCG	
	*sul1*	F: GCGCGGCGTGGGCTACCT R: GATTTCCGCGACACCGAGACAA	Same conditions, with the specific annealing temperature at 65 °C.
	*sul2*	F: CGGCATCGTCAACATAACC R: GTGTGCGGATGAAGTCAG	

**Table 5:** Type of samples used to isolate *Salmonella* spp.

Studies	Type of Samples	*Salmonella* spp. Isolates n (%)
Aslam et al. 2012 [45]	564 meat samples (206 chicken, 91 turkey, 134 beef and 133 pork)	210 isolates (183 strains from chicken; 24 strains from turkey and 3 strains from pork) (37.2%)
Dahshan et al. 2010 [46]	270 pig fecal samples	44 isolates (16.3%)
Deng et al. 2017 [38]	327 meat samples (137 pork, 91chicken and 99 beef)	252 isolates (175 strains from pork, 43 strains from chicken and 34 strains from beef) (46.5%)
Dessie et al. 2013 [27]	Chicken fecal samples	33 isolates
El-Sharkawy et al. 2017 [47]	615 samples collected from intestine, liver, and gall bladder from chickens	67 isolates (10.9%)
Hsu et al. 2014 [48]	236 water samples from river sheds	54 isolates (22.9%)
Igbinosa 2015 [44]	Cow and goat fecal samples	250 isolates (182 strains from cow feces and 68 strains from goat feces)
Iwu et al. 2016 [39]	500 adult pig fecal samples	48 isolates (9.6%)
Khoshbakht et al. 2018 [49]	Human and poultry samples	60 isolates
Kozak et al. 2009 [50]	938 chicken and swine meat samples	234 isolates (13 strains from chicken and 221 strains from swine) (24.9%)
Lapierre et al. 2010 [51]	580 healthy swine samples (290 fecal samples and 290 lymph node samples)	65 isolates (11.2%)
Lopes et al. 2015 [52]	1771 samples from pig feces and carcasses	225 isolates (12.7%)
Maka et al. 2015 [7]	Retail meat samples (poultry, pork, and beef)	84 isolates
Marquéz et al. 2017 [53]	120 hen eggshells	39 isolates (32.5%)
Mthembu et al. 2019 [54]	361 fecal samples (cattle, sheep, goats, pigs, ducks, and chickens)	106 isolates (29.4%)
Sadiq et al. 2017 [40]	Beef, poultry, and human samples	4 isolates (2 strains from human clinical samples; 1 strain from poultry and 1 strain from beef)
Soyer et al. 2013 [55]	Human and bovine samples	336 isolates (178 isolates from human and 158 isolates from bovine)
Tajbakhsh et al. 2012 [56]	1.120 samples of humans with diarrhea symptoms	71 isolates (6.4%)
Thai et al. 2012 [57]	245 pork and chicken meat shops samples (116 carcass, 84 table surfaces and 45 sewage effluent)	97 isolates (51 strains from carcass; 30 strains from table surfaces and 16 strains from sewage effluent) (39.6%)
Vital et al. 2017 [41]	410 fresh vegetables samples	24 isolates (5.85%)
Vuthy et al. 2017 [58]	762 chicken samples (80 feces, 82 chicken caeca, 440 chicken neck skins, 80 rinse water and 80 chopping boards samples selected inside chicken slaughter)	181 isolates (23.4%)
Xu et al. 2019 [10]	Agricultural samples	18 isolates
Zhu et al. 2017 [43]	627 broiler chicken samples	189 isolates (30.1%)
Zhu et al. 2019 [59]	324 pork meat samples	155 isolates (47.8%)
Zishiri et al. 2016 [42]	200 chicken samples	102 isolates (51.0%)
